# Lakes as nitrous oxide sources in the boreal landscape

**DOI:** 10.1111/gcb.14928

**Published:** 2020-01-08

**Authors:** Pirkko Kortelainen, Tuula Larmola, Miitta Rantakari, Sari Juutinen, Jukka Alm, Pertti J. Martikainen

**Affiliations:** ^1^ Finnish Environment Institute Helsinki Finland; ^2^ Natural Resources Institute Finland (Luke) Helsinki Finland; ^3^ City of Helsinki Helsinki Finland; ^4^ Faculty of Biological and Environmental Sciences University of Helsinki Helsinki Finland; ^5^ Natural Resources Institute Finland (Luke) Joensuu Finland; ^6^ Department of Environmental and Biological Sciences University of Eastern Finland Kuopio Finland

**Keywords:** climate change, ecosystems, environmental change, eutrophication, lakes, landscape, nitrous oxide, trace gases

## Abstract

Estimates of regional and global freshwater N_2_O emissions have remained inaccurate due to scarce data and complexity of the multiple processes driving N_2_O fluxes the focus predominantly being on summer time measurements from emission hot spots, agricultural streams. Here, we present four‐season data of N_2_O concentrations in the water columns of randomly selected boreal lakes covering a large variation in latitude, lake type, area, depth, water chemistry, and land use cover. Nitrate was the key driver for N_2_O dynamics, explaining as much as 78% of the variation of the seasonal mean N_2_O concentrations across all lakes. Nitrate concentrations varied among seasons being highest in winter and lowest in summer. Of the surface water samples, 71% were oversaturated with N_2_O relative to the atmosphere. Largest oversaturation was measured in winter and lowest in summer stressing the importance to include full year N_2_O measurements in annual emission estimates. Including winter data resulted in fourfold annual N_2_O emission estimates compared to summer only measurements. Nutrient‐rich calcareous and large humic lakes had the highest annual N_2_O emissions. Our emission estimates for Finnish and boreal lakes are 0.6 and 29 Gg N_2_O‐N/year, respectively. The global warming potential of N_2_O from lakes cannot be neglected in the boreal landscape, being 35% of that of diffusive CH_4_ emission in Finnish lakes.

## Graphical abstract

Up‐scaling of freshwater N_2_O emissions at regional to global scales has remained challenging due to sparse data based on summer measurements. We collected seasonal data on N_2_O concentrations from 112 randomly selected boreal lakes in Finland and determined a representative set of possible drivers. Our data underline the key role of nitrate in regulating seasonal and spatial N_2_O concentrations. Nitrate explained 78% of the variation in N_2_O across all lakes. The Global Warming Potential of N_2_O in our data was 35% of that of diffusive CH_4_ emission underlining the importance to include N_2_O in landscape GHG evasion estimates.

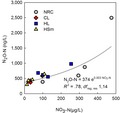

## 1 INTRODUCTION

Lakes and streams acting as recipients of carbon, nitrogen, and other nutrients transported from terrestrial ecosystems contribute to landscape greenhouse gas (GHG) balances emitting carbon dioxide (CO_2_), methane (CH_4_), and nitrous oxide (N_2_O). Freshwater N_2_O has received minor interest compared to carbon gases, CO_2_ and CH_4_. Consequently, both the variability of N_2_O concentrations and factors regulating N_2_O fluxes from lakes at regional and global scales have remained poorly constrained and estimates of freshwater N_2_O emissions are still uncertain due to sparse data (Deemer et al., 2016; DelSontro, Beaulieu, & Downing, 2018; Soued, del Giorgio, & Maranger, 2015). Majority of freshwater studies have focused on rivers and streams in N‐rich agricultural environments (Beaulieu et al., 2011; Hu, Chen, & Dahlgren, 2016; Mulholland et al., 2008) excluding landscapes dominated by forests and peatlands, the most widely distributed ecosystems in the boreal zone.

**Figure 1:**
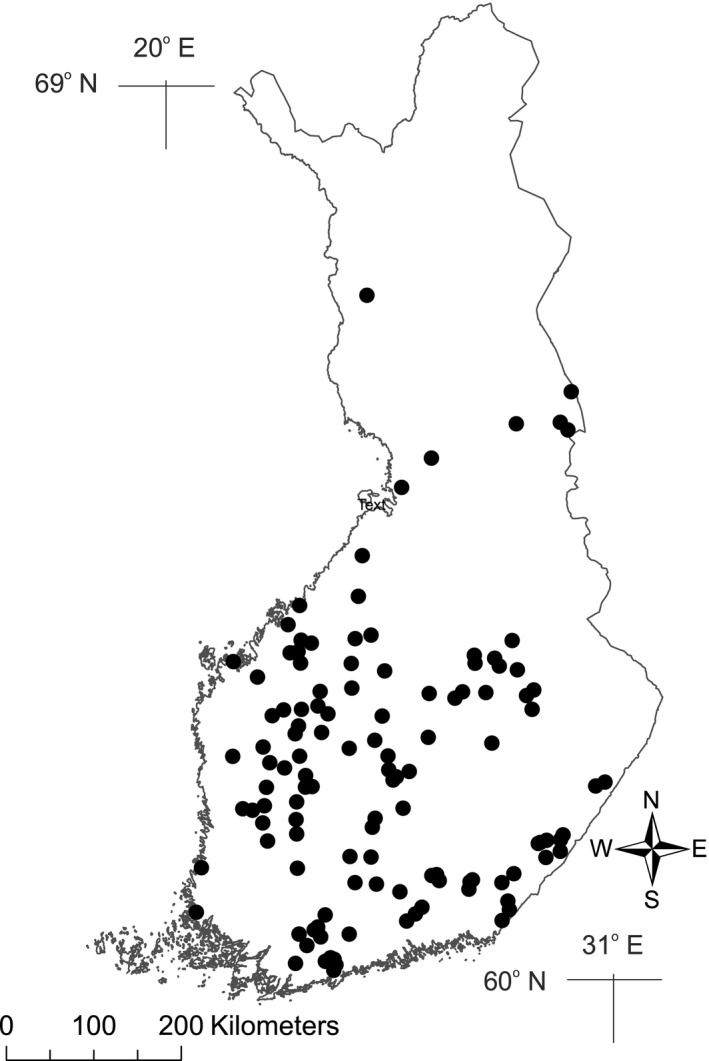
Location of the study lakes (*n* = 112)

The main processes involved in N_2_O cycling are aerobic nitrification and anaerobic denitrification, which are regulated by several environmental factors like oxygen and organic matter content, pH, temperature, and the availability of ammonium and nitrate (Butterbach‐Bahl, Baggs, Dannenmann, Kiese, & Zechmeister‐Boltenstern, 2013). In contrast to most previous studies, which have predominantly focused either on a few lakes and/or summer time measurements, we measured N_2_O concentrations for the four seasons in the water columns of 112 lakes in Finland covering different lake types, locating between the latitudes 60°N and 67°N (Figure 1; Table 1). We examined how seasonal and spatial variation of N_2_O concentrations in lakes was associated with the characteristics of lakes (area, maximum depth, water chemistry, temperature, oxygen content) and catchments (area, elevation, and land use cover) and compared spatiotemporal variation in N_2_O concentration with that of CO_2_ and CH_4_ measured simultaneously with the N_2_O (Juutinen et al., 2009; Kortelainen et al., 2006).

**Table 1:** Average catchment characteristics and lake water chemistry of autumn 1 m samples for N_2_O lakes (randomly selected lakes sampled in 1998–1999) and for Nordic Lake Survey (NLS) lakes (sampled in 1995)

	N_2_O lakes, *n* = 87				NLS, *n* = 874	
	Med	Mean	Min	Max	Med	Mean
LA (km^2^)	0.31	3.3	0.04	44	0.22	20
CA/LA	14	49	2.8	610	16	72
Max depth (m)	6.8	9.1	1	42	NM	NM
Water (%)	9.2	11	0.3	35	8.8	10
Agric. land (%)	4.2	7.6	0.03	39	2.3	5.6
Peat (%)	8.3	14	0	84	14	18
Alkalinity (µmol/L)	140	170	−70	990	130	150
pH	6.5	6.6	4.2	7.7	6.7	6.6
O_2_ (%)	82	79	47	95	NM	NM
*p*CO_2_ (µatm)	1,100	1,500	330	4,500	860	990
N_tot_ (µg/L)	500	560	170	2000	430	470
Inorganic N (%)	7.4	10	0.4	48	7.7	11
P_tot_ (µg/L)	15	21	5.0	85	14	19
Inorganic P %	15	17	1.6	63	15	19
TIC (mg/L)	2.0	2.5	0.6	11	1.6	2
TOC (mg/L)	9.4	11	2.2	38	7.8	9
Fe_tot_ (µg/L)	400	650	29	3,600	360	560

Abbreviations: CA, catchment area; LA, lake area; NM, not measured; TIC, total inorganic carbon; TOC, total organic carbon.

## 2 MATERIALS AND METHODS

### 2.1 Selection of the lakes and sampling

A subpopulation of 87 boreal lakes in Finland (Figure 1; Table 1) was randomly selected from the Nordic Lake Survey (NLS) database (Henriksen et al., 1998; Mannio, Räike, & Vuorenmaa, 2000). Besides N_2_O lake chemistry and morphometry (lake area [LA], max depth), catchment characteristics (catchment area [CA], elevation), land use cover (agricultural land %, peat %, forest %, urban %), and climate‐related variables (latitude, water temperature) were determined for each lake in order to identify key drivers contributing to seasonal variation of N_2_O concentrations in boreal lakes. The lakes were sampled once in winter, spring, summer, and autumn during 1998–1999 from four depths at the deepest point of the lake for N_2_O concentrations and physical and chemical characteristics. For CO_2_ and CH_4_ analyses, a subpopulation of 177 lakes was randomly selected from the NLS data (Juutinen et al., 2009; Kortelainen et al., 2006). Gas analyses were carried out in the laboratories of University of Eastern Finland, due to limited capacity for N_2_O analyses we had to exclude approximately one‐third of the lakes, ending up with 87 randomly selected lakes for N_2_O measurements. Majority of boreal lakes are located in forested catchments with a relatively minor human disturbance. Consequently, eutrophic lakes cannot be expected to be well represented in the randomly selected lake database. In order to include also some eutrophic lakes in the study, a subset of NLS lakes (*n* = 25) with the highest total P concentrations was also sampled. Results from these lakes were included in the analyses to study the relationships between lake water chemistry and N_2_O concentrations and flux estimates from different lake types but excluded from the population estimates.

Following similar approach as for CH_4_ we used modified Finnish lake typology required for the ecological lake status classification by the EU Water Framework Directive to classify the lakes, for example, according to nutrient concentrations, color, size, and depth (Juutinen et al., 2009). In order to avoid very small subgroups, we divided the lakes into four subgroups: Nutrient‐Rich and Calcareous (NRC, alkalinity >0.4 mmol/L, winter turbidity >5 FTU), Humic Small (HSm, color ≥30 Pt mg/L, area ≤5 km^2^, or mean depth <3 m), Humic Large (HL, color ≥30 Pt mg/L, area >5 km^2^), and Clear Water Lakes (CL, color <30 Pt mg/L).

### 2.2 Calculation of gas fluxes

N_2_O measurements were carried out by the headspace equilibration technique (McAuliffe, 1971). Ultra pure N_2_ gas (30 ml) was added to 60 ml syringes containing 30 ml water, the syringes were then shaken vigorously for 3 min. The headspace gas concentration was quantified with a gas chromatograph (Hewlett Packard Series II and Shimadzu GC‐14‐A) equipped with an FI‐detector.

Lake–atmosphere gas fluxes were estimated from the surface water gas concentrations according to the First Fick's law of diffusion (Fick, 1855; Wanninkhof & Knox, 1996):

(1) $$F_{\text{gas}}=k_{\text{gas}}\left(C_{\text{gas}}-C_{\text{eq}}\right),$$

where *F*_gas_ is the lake–atmosphere flux of N_2_O, *k*N_2_O is the gas transfer velocity (m/day), *C*_gas_ is the concentration of the gas in the surface water (μmol/L), *C*_eq_ is the concentration of the gas (μmol/L) in equilibrium with the atmosphere.

The in situ equilibrium concentration of N_2_O in the lake surface water was calculated according to Henry's Law, with appropriate corrections for the in situ temperature, assuming atmospheric concentration of 324 ppb for N_2_O (319 ppb in 2005 in Hyytiälä SMEAR station, Finland) and a 0.8 ppb increase per year (Ciais et al., 2013).

Since the data behind measured gas transfer coefficients (*k* values) are limited and the relationship between lake size and *k* values varies, we used three approaches to estimate lake–atmosphere gas exchange (Heiskanen et al., 2014; Holgerson, Farr, & Raymond, 2017; Vachon & Prairie, 2013). We calculated the gas fluxes for the 94 lakes that had N_2_O concentrations measured in all four seasons. Some lakes are located in remote areas and were difficult to sample before and/or after ice melt.

First, following the approach by Heiskanen et al. (2014), *k*N_2_O value was calculated, using the average values of *k*CO_2_ from a small Finnish lake during a 4 month period, which we transformed into the *k*N_2_O:

(2) $$k_{\text{gas}1}/k_{\text{gas}2}={\left({\text{Sc}}_{\text{gas}1}/{\text{Sc}}_{\text{gas}2}\right)}^{-n},$$

where Sc_gas_ is the Schmidt number for a given gas (Jähne, Heinz, & Dietrich, 1987) and *n* is 1/2.

The wind speed was assumed to be 3 m/s, which is an average open water period wind speed at the height of 10 m for the inland measurement stations in Finland (Leinonen, 2000).

Secondly, we applied Holgerson et al. (2017) approach for each of our 94 lakes, that is, the size‐class median estimates of *k*_600_ for 67 ponds and lakes across a size gradient measured with floating chambers or gas tracers.

Thirdly, we calculated *k*_600_ values using an empirical model based on wind speed (*U*_10_) and LA in 21 Canadian water bodies (Vachon & Prairie, 2013):

(3) $$k_{600}=2.51+1.48\;U_{10}+0.39\;U_{10}\;\log_{10}\;\text{LA}.$$

### 2.3 Estimation of annual fluxes and upscaling

First, following similar approach as for CO_2_ (Kortelainen et al., 2006), we estimated the annual N_2_O emission per surface area unit of each lake using the N_2_O concentrations from the depth of 1 m during the four sampling occasions as follows: the winter 1 m concentrations were used for half a month period assuming that the N_2_O accumulated under ice would be released to the atmosphere during the short spring circulation period. The spring N_2_O concentrations at the depth of 1 m were used for a 1.5 month period and the summer 1 m concentrations for a 3 month period representing the summer stratification between May and August. The samples taken during the autumn circulation were used for the period of 2 months representing the time after breakup of the summer stratification and before the permanent ice cover (altogether 7 months of ice‐free season). For comparison, we also estimated the evasion for the ice‐free period by multiplying the median summer time N_2_O flux with the duration of the ice‐free period (7 months).

Secondly, we estimated the annual median N_2_O evasion both for each lake size class separately (lake size‐specific evasion) and for all size classes combined (the mean, median, and summer median of individual lakes) for Finnish and Boreal lakes. The randomly selected lakes, which could be sampled during all four seasons (*n* = 71), were divided into the different size classes and the median annual evasion of each size class was multiplied with the area of the Finnish and boreal lakes belonging to the respective size class. Summing up the size class‐specific estimates results in an estimate for Finland and boreal region, respectively. The rationale was that when upscaling is based on the evasion estimated specific to each of the four lake size classes, the impact of the numerous small lakes in our data on the regional estimate is not disproportionally large.

Furthermore, we estimated the contribution of N_2_O emission from lakes to that of forests both in Finland and in the boreal zone. For Finland, we used the lake surface area of 32,663 km^2^ (Raatikainen & Kuusisto, 1990) and the forest area of 0.203 × 10^6^ km^2^ (Vaahtera et al., 2018). For the boreal region, the lake surface area was estimated from MODIS data (1,422,448 km^2^), the forest area of 12.1 × 10^6^ km^2^ was derived from Potter, Matson, Vitousek, and Davidson (1996).

### 2.4 Water chemistry

The lakes were sampled in each sampling occasion for dissolved oxygen, alkalinity, conductivity, pH, color, total nitrogen (N_tot_), nitrate (+ nitrite) nitrogen defined as (NO_3_‐N, nitrite had negligible contribution to the total amount of nitrate and nitrite), ammonium nitrogen (NH_4_‐N), total phosphorus (P_tot_), phosphate phosphorus (PO_4_‐P), total organic carbon (TOC), and total iron (Fe_tot_). Water chemistry was analyzed from unfiltered samples in the accredited laboratories of the Regional Environment Centers. N_tot_ was determined by oxidation with K_2_S_2_O_8_, reduction of NO_3_‐N to NO_2_‐N in Hg‐Cd (Cu‐Cd) column and colorimetric determination of azo‐color. The sum of NO_3_‐N and NO_2_–N was measured by reduction of NO_3_‐N to NO_2_‐N in Hg‐Cd (Cu‐Cd) column, followed by colorimetric determination of azo‐color. NH_4_‐N was measured colorimetrically with hypochlorite and phenol. P_tot_ and PO_4_‐P were measured colorimetrically. TOC was determined by oxidizing the sample by combustion and measuring inorganic C by IR‐spectrophotometry (National Board of Waters, 1981).

### 2.5 Catchment characteristics

The CAs of the NLS lakes were determined from the topographic maps, and the catchment boundaries were digitalized and combined with land use data based on satellite images using the Arc View georeferencing software. Lake area, CA, catchment to LA ratio, latitude, and the proportion of peatland, forest on mineral soil, agricultural land, water (consisting of the upstream water bodies and the lake itself), and built‐up area in the catchments were determined.

### 2.6 Statistical analysis

The relationships between the N_2_O concentrations and lake chemistry, morphometry, latitude, and catchment characteristics were examined using Pearson's correlation coefficients using SAS 9.4 for Windows software. The variables were log_e_ or square root transformed in order to normalize their distribution.

Stepwise multiple linear regression models predicting N_2_O concentrations were carried out using lake chemical, physical, and morphometric variables, climatic variables (temperature, latitude), and catchment properties as predictors. The cases with an absolute value of the studentized residual exceeding 3 were excluded and only the independent variables with *p* < .05 were included in the models.

Linear mixed models were used to take into account that each lake was sampled four times and from four depths (interdependence of within‐lake sampling). We run a linear mixed model with NO_3_‐N, depth, season as fixed factors, and lake as random factor. That is, the model analyzed the dependence of N_2_O concentration on NO_3_‐N, and eliminated the effects of depth and season on the emerging N_2_O to NO_3_‐N relationship.

To examine the relationship between mean NO_3_‐N and mean N_2_O concentrations in different lake types across the four seasons, a nonlinear regression model was fitted (Equation 4, see Results). Seasonal mean values for each lake type (*n* = 16) were obtained by averaging all measured depths across respective lakes. To examine how representative the patterns and drivers of N_2_O dynamics found in our lakes were, we used Equation (4) to estimate N_2_O concentrations in the water samples from the independent nitrate‐nitrite dataset of 874 randomly selected Finnish NLS lakes sampled during the autumn overturn in 1998–1999. The modeled results were compared to the measured N_2_O‐N concentrations in our study lakes (*n* = 1,542 seasonal water samples from 112 lakes).

## 3 RESULTS

### 3.1 Seasonal and spatial variation in N_2_O

**Figure 2:**
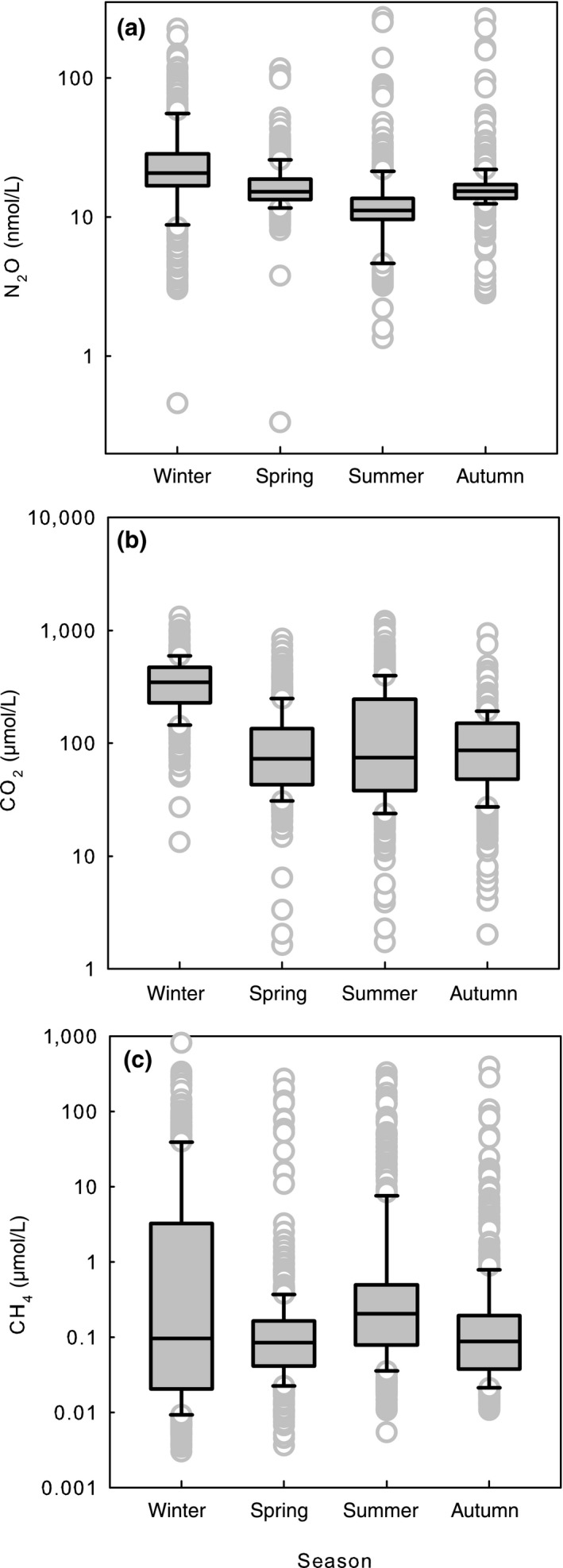
Seasonal distribution (median, first and third quartile) of the concentrations of N_2_O (*n* = 87) (a), CO_2_ (*n* = 177) (b), and CH_4_ (*n* = 177) (c), in randomly selected lakes, all depths. Minimum N_2_O concentrations were measured in summer in contrast to CO_2_ and CH_4_ distributions. Concentrations of CO_2_ and CH_4_ are based on the data from Kortelainen et al. (2006) and Juutinen et al. (2009). Note that *y*‐axis is on a log scale

During the open water season, 71% of the surface water samples were saturated with respect to the atmospheric equilibrium value of N_2_O, that is, lakes were mostly sources of N_2_O. Nitrous oxide, similar to CO_2_, peaked in winter. In contrast, CH_4_ concentration was significantly higher in summer (Kruskal–Wallis 123.93, *p* < .001, Figure 2).

**Figure 3:**
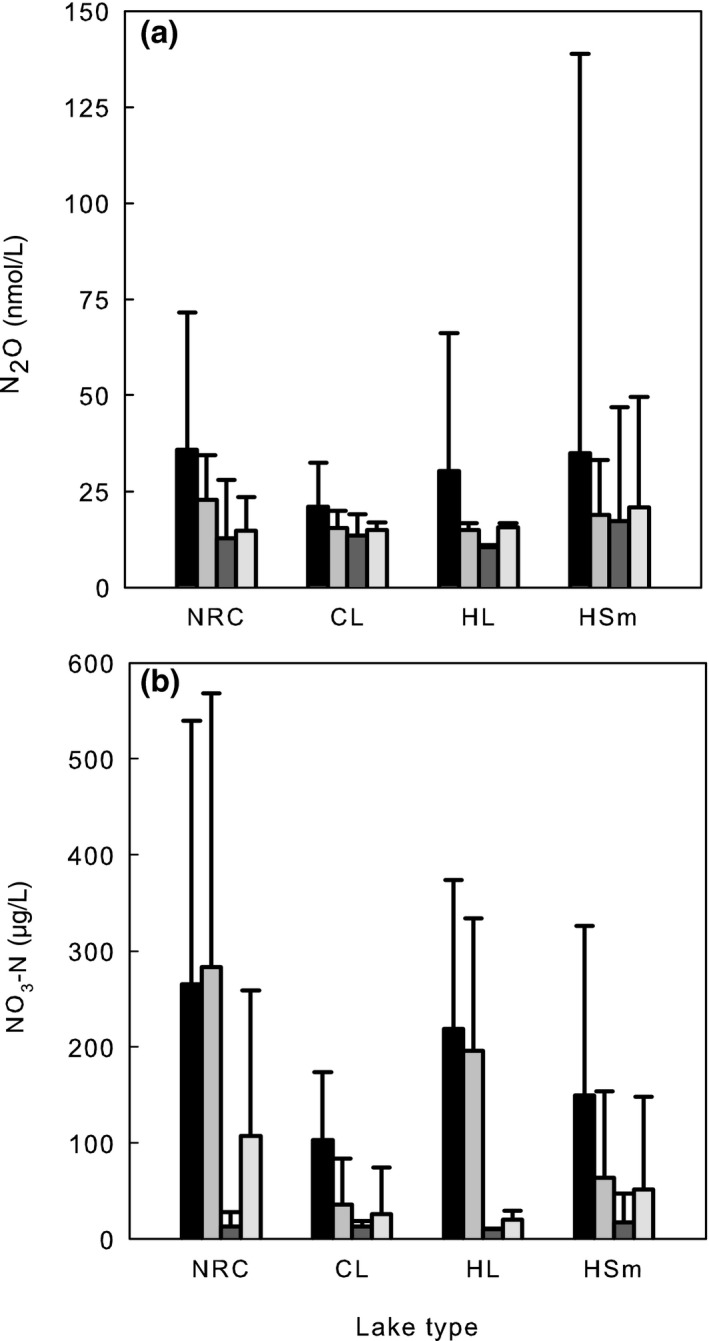
Distribution of N_2_O (a) and NO_3_‐N (b) across different lake types, randomly selected lakes, all seasons and depths. NRC, Nutrient‐Rich and Calcareous; HSm, Humic Small; HL, Humic Large; and CL, Clear Water Lakes. Seasonal variation of N_2_O concentrations followed the variation of nitrate—highest in winter and lowest in summer despite differences in concentration levels across the lake type classification

**Figure 4:**
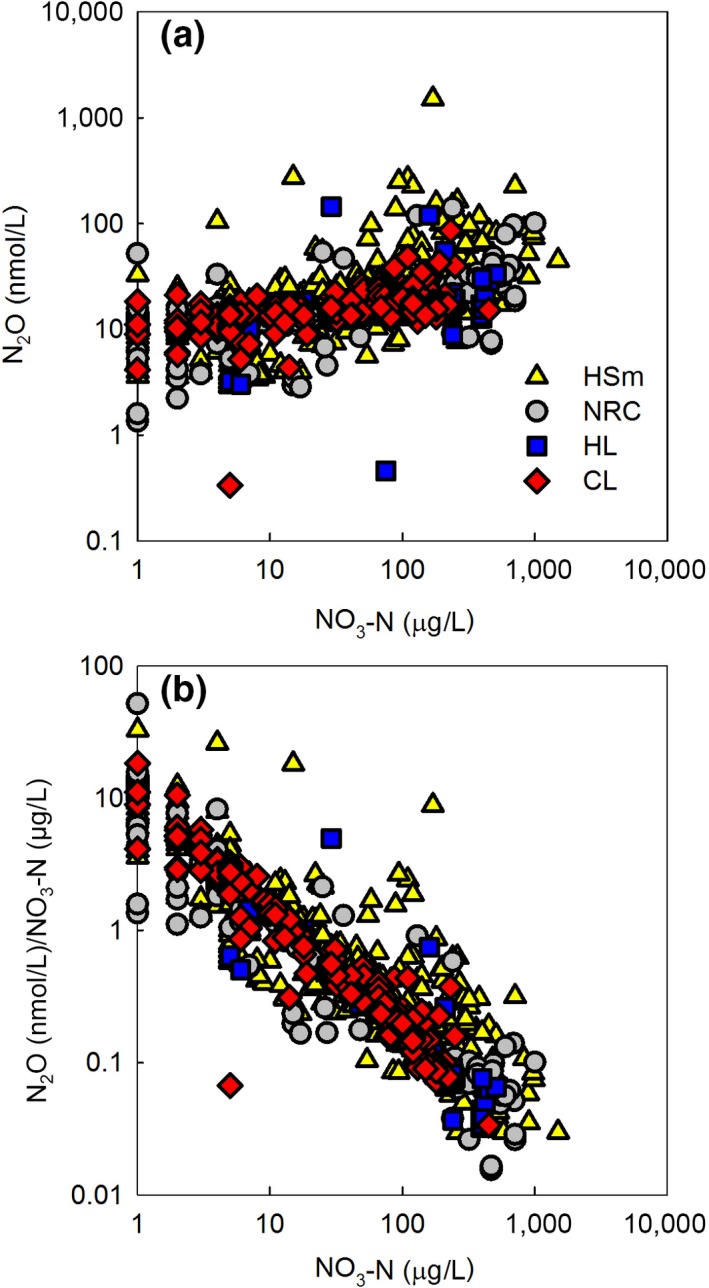
The relationship between NO_3_‐N and N_2_O (a), and NO_3_‐N and N_2_O/NO_3_‐N ratio (b). All lakes, seasons, and depths. Lake type identified, all data were log‐transformed. Our data across all lake types and seasons showed strong positive correlation between nitrate and N_2_O and strong negative correlation between nitrate and N_2_O/NO_3_‐N ratio

**Figure 5:**
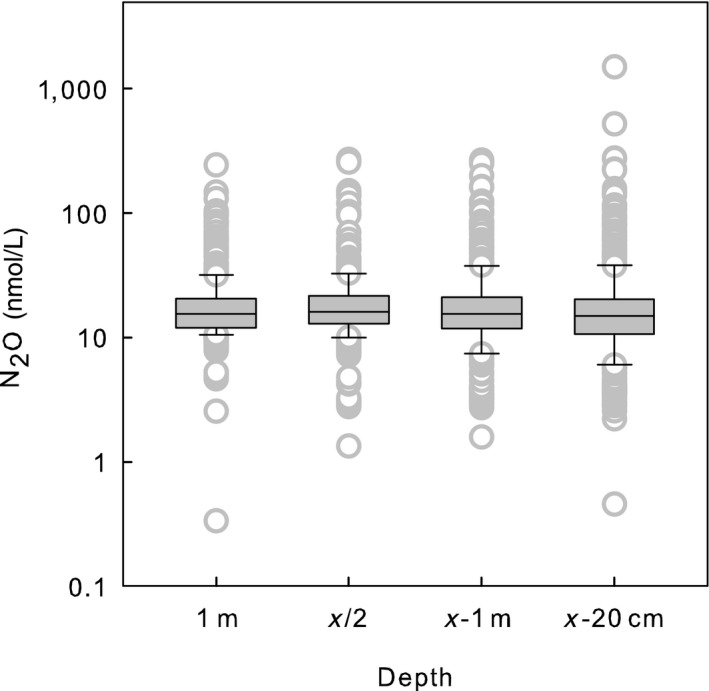
Distribution (median, first and third quartile) of N_2_O across depth; randomly selected lakes, all seasons. Median N_2_O was evenly distributed across depth

Seasonal variation of N_2_O concentrations followed the variation of nitrate—highest in winter and lowest in summer despite differences in concentration levels across lake type classification (Figure 3). Furthermore, nitrate was the best predictor for N_2_O concentrations in the entire data (Table 2; Figure 4) and across different seasons and depths despite the large variation in LA (from 0.04 to 44 km^2^), maximum depth (from 1 to 42 m), latitude (from 60°N to 67°N), and land use cover. Nitrate concentrations were highest in nutrient‐rich, calcareous (NRC) lakes accompanied by highest N_2_O concentrations in winter and spring (Figure 3). In summer and autumn, N_2_O concentrations were more evenly distributed across the lake types. Depth profile distribution demonstrated similar N_2_O concentrations from surface to bottom (Figure 5) in contrast to CO_2_ and CH_4_ which accumulated in bottom water (Juutinen et al., 2009; Kortelainen et al., 2006).

**Table 2:** Correlation coefficient values between N_2_O and lake (area) and catchment area, maximum depth, latitude, land use cover, elevation, water temperature, and water chemistry in all lakes (randomly selected and eutrophic lakes), in surface water (all seasons), in bottom (all seasons), and in winter (all depths)

	All data	Surface	Bottom	Winter
ln LA	0.183***	NS	0.248***	0.140*
ln CA	0.294***	0.154*	0.342***	0.267***
ln Maximum depth	0.076*	NS	NS	NS
Lake latitude	−0.136***	−0.194***	−0.139*	−0.228***
Forest %	NS	0.100*	NS	NS
√Peat %	NS	−0.123*	NS	−0.155**
√Field %	0.211***	0.232***	0.190***	0.345***
√Built‐up %	NS	NS	NS	0.168**
√Water %	−0.168***	−0.209***	−0.102*	−0.215***
Lake elevation	−0.195***	−0.158***	−0.193***	−0.334***
Water temperature	−0.378***	−0.558***	−0.304***	−0.239***
ln N_tot_	0.0692*	0.332***	NS	NS
ln NH_4_	NS	0.377***	NS	−0.178**
ln NO_3_	0.593***	0.582***	0.612***	0.635***
ln P_tot_	NS	0.194***	NS	0.141*
ln PO_4_	NS	0.381***	NS	0.115*
ln TOC	0.0737*	0.110*	NS	NS
ln Water color	NS	0.147*	NS	NS
ln O_2_	0.189***	−0.105*	0.206***	0.276***
ln Fe_tot_	NS	0.192***	NS	NS
ln Conductivity	0.148***	0.260***	NS	0.210***
ln Alkalinity	NS	0.143*	NS	NS
pH	NS	−0.120*	NS	NS

Abbreviations: CA, catchment area; LA, lake area; NS, not significant; TOC, total organic carbon.

*p < .05; **p < .01; ***p < .001.

**Figure 6:**
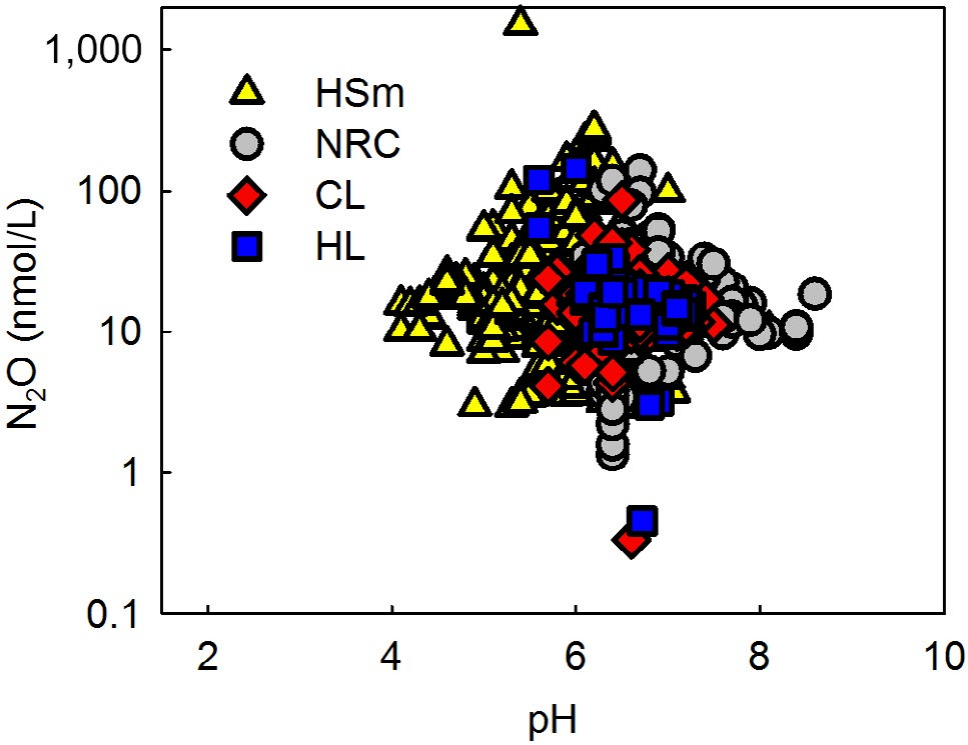
N*_2_*O distribution across pH, different lake types identified as in Figure 3; all lakes, depths, and seasons. Both the highest and the lowest N_2_O concentrations occurred around the median pH of 6.5

**Figure 7:**
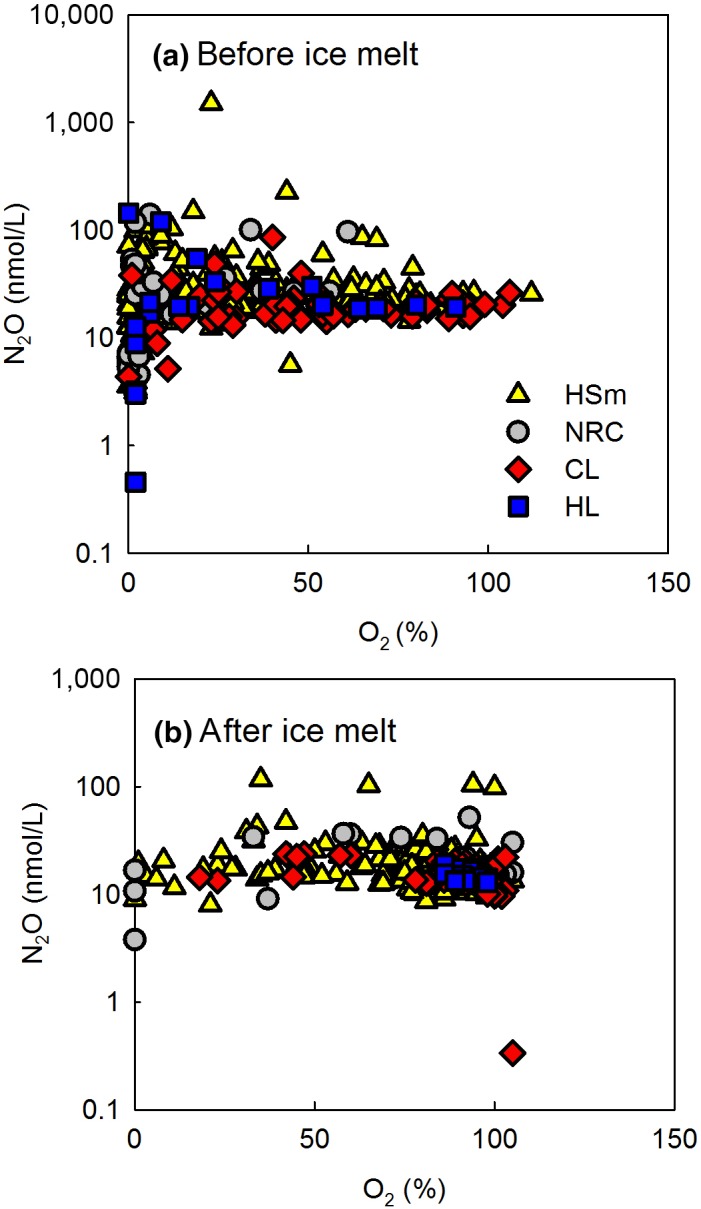
N*_2_*O concentrations in different lake types before (a) and after ice melt (b) across oxygen saturation percentage; all lakes and depths. Oxygen was not a key driver for N_2_O in Finnish boreal lakes

**Figure 8:**
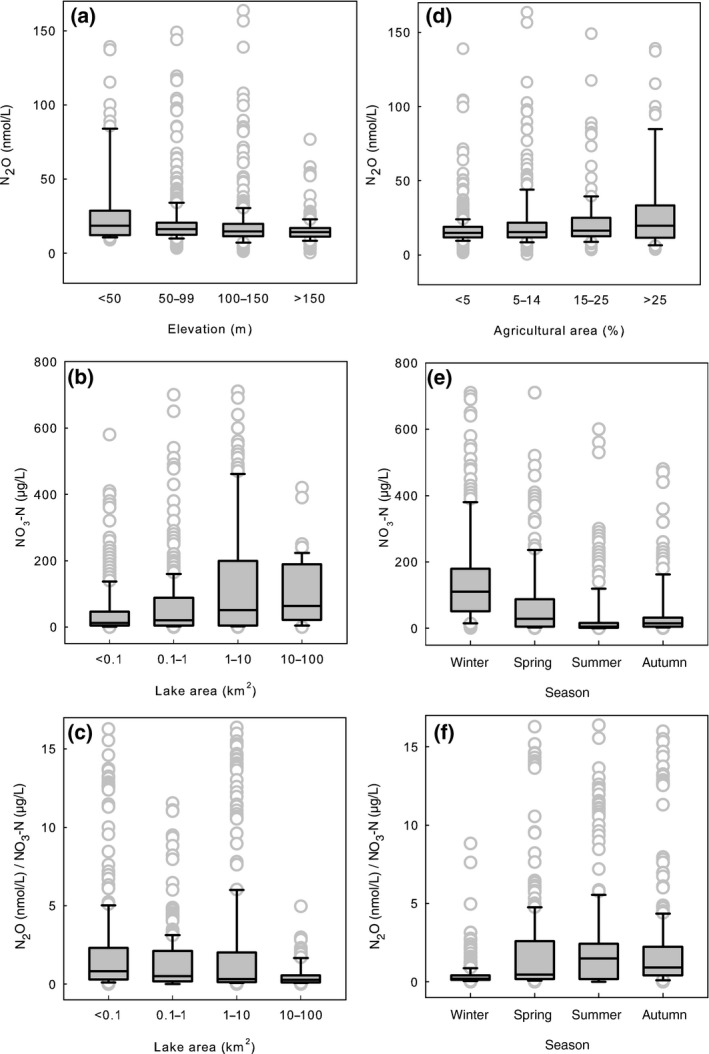
Distribution (median, first and third quartile) of N_2_O across elevation (a) and the percentage of agricultural land in the catchment (d). Distribution (median, first and third quartile) of NO_3_‐N across lake area (LA) (b) and season (e). Distribution (median, first and third quartile) of N_2_O/NO_3_‐N ratio across LA (c) and season (f), randomly selected lakes, all depths

There was no district correlation between N_2_O and water pH. Both the highest and the lowest N_2_O concentrations occurred around the median pH of 6.5 (Figure 6). Even though there was a weak correlation between oxygen and N_2_O (Table 2), the variability in N_2_O concentrations across O_2_ gradient was large especially before ice melt among all lake types (Figure 7). N_2_O and nitrate concentrations increased with decrease in elevation (Figure 8a) accompanied by increasing coverage of agricultural land (Figure 8d), which resulted in higher nitrate and N_2_O concentrations in large lakes (Figure 8b) often surrounded by more intensive human impact compared to small headwater lakes, which in the boreal zone are predominantly surrounded by forests and peatlands.

Lake chemistry predicted N_2_O better than catchment land use cover. In linear multiple regression models, electron acceptors, nitrate and oxygen, and lake water temperature as independent variables predicted best (*r* ^2^ = .55, stepwise procedure) the N_2_O in the entire data (*n* = 1,396, all seasons and depths). Nitrous oxide in bottom water (all seasons) was best predicted by nitrate and oxygen concentration (*r* ^2^ = .54). The surface water model (all seasons) had nitrate, temperature, and the percentage of agricultural land in the catchment as the independent variables explaining 58% of the variation in N_2_O. The best model for winter (all depths) explained 58% of the variability in N_2_O selecting nitrate, latitude, and pH as the independent variables (Table 3). The linear mixed model results demonstrated that the significant relationship between nitrate and N_2_O remained (*p* < .001) even after the influence of depth (*p* = .096) and season (*p* < .001) had been taken into account.

**Table 3:** Selected linear stepwise multiple regression equations for N_2_O (water chemistry, land use cover, climatic drivers, and catchment topography were used as predictors; randomly selected and eutrophic lakes)

Dataset	*n*	Dependent variable		Intercept		Parameter estimate		Parameter	Unit	Model *R* ^2^
All data	1,396	ln N_2_O	=	2.31	+	0.145	×	ln NO_3_‐N	µg/L	.41
						0.128	×	ln O_2_	%	.51
						−0.0303	×	Temperature	°C	.55
Surface	397	ln N_2_O	=	2.74	+	0.0952	×	ln NO_3_‐N	µg/L	.47
						−0.0373	×	Temperature	°C	.56
						0.0377	×	√Field %	%	.58
Bottom	408	ln N_2_O	=	2.07	+	0.258	×	ln NO_3_‐N	µg/L	.46
						0.496	×	ln O_2_	%	.54
Winter	379	ln N_2_O	=	123	+	0.394	×	ln NO_3_‐N	µg/L	.52
						−7.68	×	ln Latitude	°N	.57
						−0.191	×	pH		.58

### 3.2 N_2_O evasion

We estimated the median N_2_O evasion based on 71 lakes (<100 km^2^) as 0.009 g N m^−2^ LA year^−1^. The seasonal median fluxes were 0.002 g N/m^2^ LA at the thaw (during 0.5 months), 0.002 g N/m^2^ LA in spring (1.5 months), 0.001 g N/m^2^ LA in summer (3 months), and 0.003 g N/m^2^ LA in autumn (2 months). For the largest lakes (>100 km^2^), we used the evasion estimate from the 10–100 km^2^ lake size class.

The median evasion for the <0.1, 0.1–1, 1–10, 10–100 km^2^ lake size classes was estimated as 0.0047, 0.007, 0.018, and 0.02 g N/m^2^ LA, respectively (using *k* values from Holgerson et al., 2017). Nitrous oxide data were not available for lakes larger than 100 km^2^; for this lake size class, we used the median evasion estimate from the 10–100 km^2^ size class. N_2_O evasion per surface area unit was highest in the largest lakes reflecting the distribution of nitrate concentrations. In contrast, CO_2_ evasion estimates per surface area unit (Kortelainen et al., 2006) were largest in small lakes.

**Figure 9:**
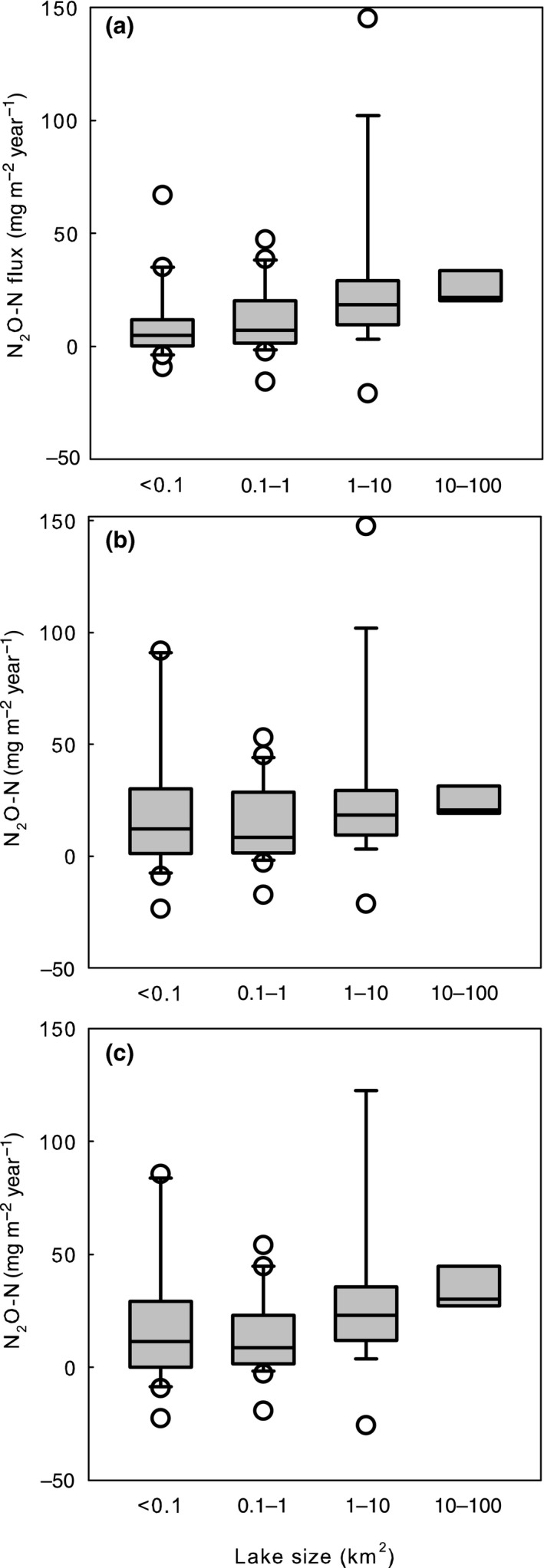
Estimated annual N_2_O flux to the atmosphere across different lake size classes; randomly selected lakes (*n* = 71) by the approaches of Holgerson et al. (2017) (a), Heiskanen et al. (2014) (b), and Vachon and Prairie (2013) (c). Nitrate and N_2_O concentrations were largest in large lakes resulting in largest emission estimates per surface area unit from largest lakes in contrast to CO_2_ and CH_4_

Total annual N_2_O flux from Finnish lakes (total LA 32,663 km^2^) was estimated as 0.6–0.8 Gg N_2_O‐N/year, based on the areas of the lake size distribution by Raatikainen and Kuusisto (1990) and the evasion estimates for the different lake size classes (Figure 9). Both Holgerson et al. (2017) and Heiskanen et al. (2014) approaches resulted in an estimate of 0.6 Gg N_2_O‐N/year for Finnish lakes, when the median N_2_O evasion estimates of different lake size classes were multiplied with the respective lake surface area distribution. The Vachon and Prairie (2013) approach resulted in a little bit larger estimate of 0.8 Gg N_2_O‐N/year (Table 4; Tables S1 and S2).

**Table 4:** Estimates of annual N_2_O flux (Gg N_2_O‐N/year) from Finnish and Boreal lakes. Annual estimates were calculated from our randomly selected set of lakes (*n* = 71) using *k* values from Holgerson et al. (2017) and different upscaling approaches: multiplying the area of the Finnish and boreal lakes with N_2_O flux estimates for each lake size class and for all size classes combined (the mean, median, and summer median of individual lakes). For the median flux, the 25% and 75% quartiles and for the mean flux 95% confidence intervals, respectively, are given in parentheses. Two small humic lakes with fluxes of 863 and 22,085 mg N_2_O‐N m^−2^ year^−1^ were excluded as outliers

Upscaling approach	Finnish Lakes	Boreal lakes
Median flux by lake size class	0.6 (0.5–0.7)	29 (26–32)
Median flux of individual lakes	0.3 (0.09–0.7)	12 (4–31)
Summer median flux of individual lakes	0.07 (−0.2 to 0.3)	3 (−10 to 13)
Mean flux of individual lakes	0.5 (0.3–0.7)	23 (15–31)
Area (km^2^)	32,663^a^	1,422,448^b^

a Lake area distribution (Raatikainen and Kuusisto, 1990).

b Lake area distribution estimated based on MODIS data, excluding lakes <0.1 km^2^.

**Figure 10:**
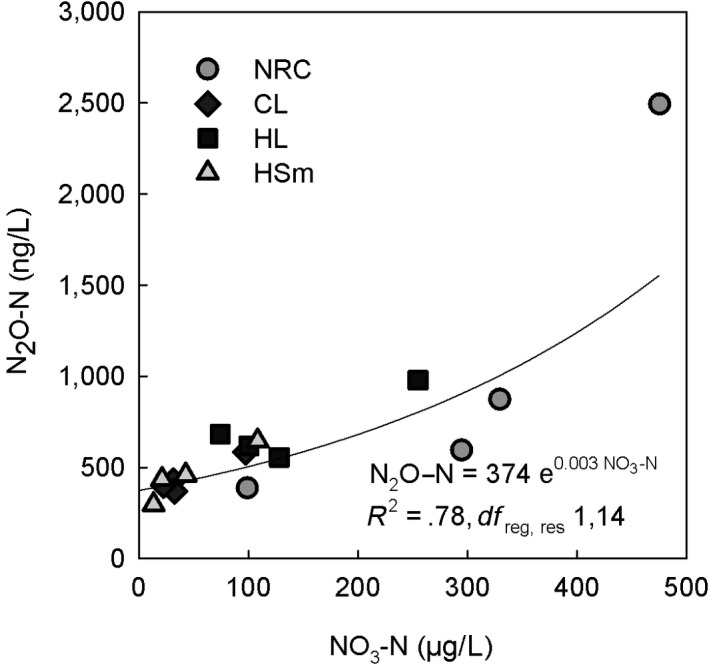
The relationship between mean NO_3_‐N and mean N_2_O concentrations in different lake types across the four seasons (Equation 4). Seasonal mean values for each lake type (*n* = 16) were obtained by averaging all measured depths across respective lakes. Our seasonal data from 112 boreal lakes in Finland underline the key role of nitrate in regulating seasonal N_2_O concentrations

Freshwater N cycling integrates numerous simultaneous temperature‐dependent microbiological processes. Also, our data demonstrated large differences in N_2_O concentrations and estimated emissions among different lake types (Table 5; Table S2). Nevertheless, our data underline the key role of nitrate in regulating seasonal and spatial N_2_O concentrations across boreal lakes. Nitrate explained as much as 78% of the variation in seasonal mean N_2_O concentrations across all lakes and depths (Equation 4; Figure 10). While O_2_ was the dominating driver for CO_2_ in our data, with similar explanation power, 78%, of the variation across all lakes and depths (Kortelainen et al., 2006).

(4) $$N_{2}O\;-\;N\left(\text{ng}/L\right)=374\;e^{0.003\;{\text{NO}}_{3}\;-\;N\left(\mu g/L\right)}$$

**Table 5:** Annual N_2_O flux estimates (mg N_2_O‐N m^−2^ year^−1^) by lake type based on all lakes that were sampled at all four seasons (*n* = 94; the water quality data were missing from two lakes and the lake type could not be assigned): annual fluxes for the randomly selected lakes (*n* = 71) and for the subset of Eutrophic lakes with the highest total P concentrations (*n* = 23). The annual fluxes (7 month ice‐free season) consist of fluxes at the thaw (0.5 months), in spring (1.5 months), in summer (3 months), and in autumn (2 months) calculated using *k* values by Holgerson et al. (2017). Two small humic lakes with fluxes of 863 and 22,085 mg N_2_O‐N m^−2^ year^−1^ were excluded as outliers

Lake type/group	Mean	Median	*SD*	CV%	*N*
Nutrient‐rich, calcareous	43	35	33.1	78	12
Clear water	9	11	11.7	126	14
Humic, large	60	41	44.2	74	7
Humic, small	18	8	29.7	164	59
All	23	11	32.2	142	94
Randomly selected	16	9	25.1	155	71
Eutrophic	43	35	42.6	99	23

**Figure 11:**
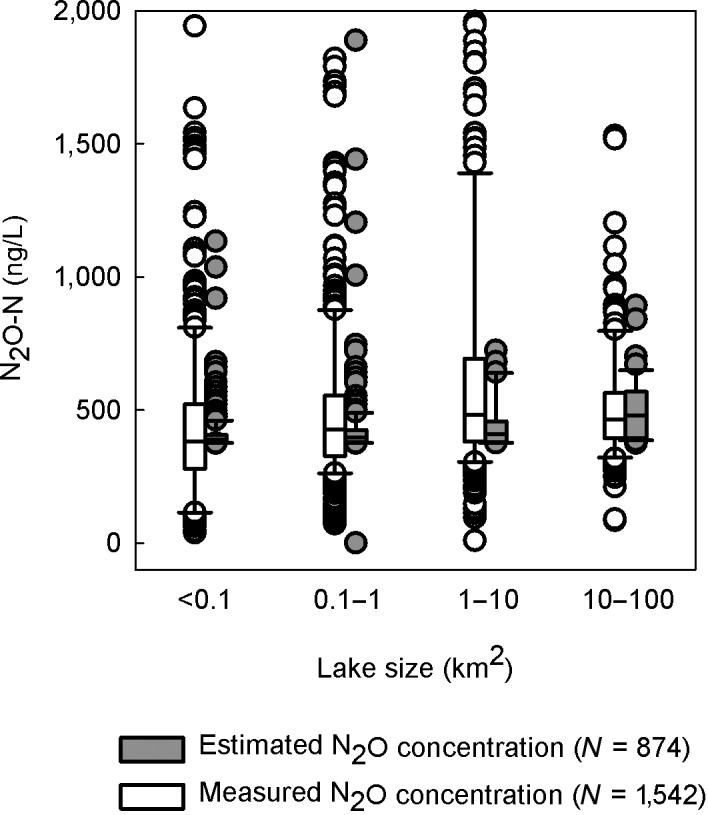
Estimated N_2_O‐N concentrations (by Equation 4) in the Finnish NLS lakes (*n* = 874 autumn 1 m samples; gray symbols; see Table 1). The measured N_2_O‐N concentrations in our study lakes (*n* = 1,542 seasonal water samples in 112 lakes) are shown for comparison (white symbols). The developed equation between nitrate and N_2_O well predicted N_2_O‐N in the data of 874 randomly selected Finnish lakes sampled in autumn 1995 (NLS lakes)

Using Equation (4), the modeled N_2_O concentrations for independent nitrate‐nitrite dataset of 874 randomly selected Finnish NLS lakes reproduced similar median N_2_O concentrations and an increasing trend by lake size further indicating that our lake dataset is representative to Finnish conditions and can be expected to represent lakes over larger boreal landscape (Figure 11).

Large lakes turned out to be disproportionately important N_2_O sources among the lake population on the landscape level. Lakes larger than 10 km^2^ were estimated to contribute 77% of the total N_2_O emission from Finnish lakes. In contrast, CO_2_ evasion estimates demonstrated that lakes smaller than 10 km^2^ dominated landscape CO_2_ evasion among the lake population while lakes larger than 10 km^2^ (representing 65% of the total LA distribution) represented only 45% of the estimated CO_2_ evasion (Kortelainen et al., 2006). Our estimated N_2_O‐N emissions from lakes represent 17% of the N_2_O‐N emissions from boreal forests, the dominating ecosystem in Finland where lakes cover 10% of the total land area (Table 6).

**Table 6:** Estimates of annual N_2_O flux from Finnish and boreal lakes (calculated as median fluxes by lake size class, *k* values by Holgerson et al. (2017) compared to estimates of annual N_2_O flux from boreal forest in Finland and in boreal zone (using the estimate of 17.6 mg N_2_O‐N m^−2^ year^−1^; Potter et al., 1996)

	Finland	Boreal region
Lake N_2_O‐N flux (Gg/year)	0.6	29
Forest N_2_O‐N flux (Gg/year)	4^a^	213^a^
LA (km^2^)	32,663^b^	1,422,448^d^
Forest area (km^2^)	203,000^c^	21,200,000^a^
Lake N_2_O‐N flux/Forest N_2_O‐N flux (%)	17	14

Abbreviation: LA, lake area.

a Potter et al. (1996).

b Raatikainen and Kuusisto (1990).

c Vaahtera et al. (2018 **)**.

d Lake area distribution estimated based on MODIS data, excluding lakes <0.1 km^2^.

## 4 DISCUSSION

### 4.1 Seasonal and spatial variation in N_2_O concentrations and fluxes

N_2_O concentrations were high in lakes located in low elevation catchments accompanied by large coverage of agricultural land (Figure 8a,d). Nutrient‐rich, calcareous lakes had high N_2_O concentrations, which were comparable with methane distribution (Juutinen et al., 2009) and underlines close links between lake trophic state and GHG concentrations, further suggesting increasing GHG evasion as a result of eutrophication. Although calcareous lakes had high N_2_O concentrations, correlation between pH and N_2_O was negligible (Table 2; Figure 6). The variability in N_2_O concentration was the largest around the median pH 6.5 (Figure 6), which may reflect optimal pH of the accumulation of N_2_O, that is, net production of N_2_O from nitrification and denitrification. Low pH inhibits the N_2_O reductase which increases the N_2_O to N_2_ ratio in denitrification (Richardson, Felgate, Watmough, Thomson, & Baggs, 2009). Nitrification is further inhibited at high C:N ratios (Her & Huang, 1995), typical for boreal Finnish lakes and often accompanied by low pH (Kortelainen et al., 2013). Supportingly, Humic large lakes, including only seven lakes in our dataset, also had high N_2_O concentrations (Figures 3 and 10).

Nitrous oxide peaked in winter similar to CO_2_, while CH_4_ concentration was significantly higher in summer (Figure 2). During the open water season, 29% of the surface water samples were under saturated with respect to the atmospheric equilibrium value of N_2_O showing that lakes can also act as N_2_O sinks, which supports results from Canadian freshwaters (Soued et al., 2015) and farm water bodies in the United States (Webb et al., 2019) underlining the uncertainty in the data presently used both in regional and global N_2_O budgets. On average, N_2_O concentrations were evenly distributed through the water column (Figure 5), which contrasts the vertical distribution patterns of CO_2_ and CH_4_ that accumulate above the sediment (Juutinen et al., 2009; Kortelainen et al., 2006). Comparable distribution of GHGs has been reported from 13 temperate lakes in Colorado Rocky Mountains (McCrackin & Elser, 2011).

Generally, N_2_O production is limited by low N turnover and low N mineralization in the high latitude N limited ecosystems (Potter et al., 1996). In N limited boreal terrain, N turnover is rapid and internal N cycling across forested ecosystems dominates the spatial nitrate distribution, which often reflects more closely catchment land use cover and topography than N deposition—in spite of N deposition being the major N source (Palviainen, Lehtoranta, Ekholm, Ruoho‐Airola, & Kortelainen, 2015). Nitrate is rapidly captured during growing season resulting in minor nitrate concentrations in downstream lakes in summer (Rekolainen, Mitikka, Vuorenmaa, & Johansson, 2005).

The N_2_O/NO_3_‐N ratio correlated negatively with nitrate (Figure 4b) being lowest in large lakes (Figure 8c) which had higher nitrate content than the small lakes. Increase in nitrate availability favors denitrification rate in lakes, but the efficiency of denitrification declines with increasing N inputs (Mulholland et al., 2008; Rissanen, Tiirola, Hietanen, & Ojala, 2013). Seasonal variation in N_2_O/NO_3_‐N ratio was also strong—highest in summer and lowest in winter (Figure 8f). Low temperature decreases the activity of N_2_O reductase more than the activity of other reductases in denitrification (Veraart, de Klein, & Scheffer, 2011) which together with high nitrate availability favors N_2_O accumulation in the dormant season. The possible higher reduction of N_2_O to N_2_ as a result of low oxygen content (Richardson et al., 2009) in dormant season did not prevent N_2_O accumulation (Figure 7). In summer, low nitrate content and high N_2_O/NO_3_‐N ratio resulted from nitrate being consumed in primary production, denitrification, and other microbial processes. In freshwaters, 0%–4% of N is generally released as N_2_O in denitrification (Mulholland et al., 2008; Seitzinger, 1988; Silvennoinen, Liikanen, Torssonen, Stange, & Martikainen, 2008).

The explanation power of our statistical models for N_2_O concentrations in lakes (Table 3) is comparable to the power of the models developed for terrestrial N_2_O emissions (Leppelt et al., 2014; Pärn et al., 2018). In the lake dataset from boreal southern Norway and Sweden, N_2_O concentrations correlated positively with nitrate in summer (Yang et al., 2015). In contrast, association between nitrate and N_2_O has been weak in temperate lakes. No significant correlation between nitrate and N_2_O could be found across temperate headwater Irish lakes in summer (Whitfield, Aherne, & Baulch, 2011). Furthermore, N_2_O fluxes could not be predicted by any measured environmental variables in aquatic network in temperate Quebec, Canada, where 40% of the relatively pristine inland waters were undersaturated in N_2_O in summer (Soued et al., 2015).

### 4.2 Landscape scale patterns

Key processes and feedbacks of landscape scale GHG fluxes have remained poorly quantified. Dynamics of N_2_O in our lakes at landscape scale did not follow those of CO_2_ (Kortelainen et al., 2006) and CH_4_ (Juutinen et al., 2009; Figure 1). Large lakes dominate the lake surface area distribution in Finland (Raatikainen & Kuusisto, 1990). Furthermore, estimated N_2_O emissions per surface area unit were largest from large lakes (Figure 9) reflecting the distribution of nitrate concentrations. In contrast, concentrations and estimated emissions of CO_2_ and CH_4_ decrease with decrease in LA (Denfeld, Kortelainen, Rantakari, Sobek, & Weyhenmeyer, 2016; Juutinen et al., 2009; Kortelainen et al., 2006).

Our results underscore simultaneous (Miettinen et al., 2015) long‐term CO_2_, CH_4_, and N_2_O measurements from freshwaters in order to better understand major controls of landscape GHG evasion. Freshwater GHG flux measurements have predominantly been carried out in summer, while our study underscores the importance of dormant season N_2_O measurements. The lower the temperature the higher N_2_O concentrations, which in our data underline the link between seasonal variability of nitrate and N_2_O concentrations, that is, lower concentrations in warm growing season compared to cold dormant period. Elevated N_2_O emissions during winter snow cover period have been measured also, for example, in cropland (Groffman et al., 2001). Higher N_2_O emission was shown to coincide with a greater number of freeze–thaw cycles that broke up soil macro aggregates and increased soil inorganic N pool (Ruan & Robertson, 2017). In a northern hardwood forest reduced N uptake by fine roots due to soil freezing was concluded to be the primary regulator of increasing nitrate export. Increasing nitrate and N_2_O concentrations during dormant period might thus ultimately reflect declining N uptake in terrestrial ecosystems resulting in increasing nitrate concentrations in downstream freshwaters (Campbell, Socci, & Templer, 2014). Freeze–thaw‐related N_2_O fluxes were shown to be a major component of annual N_2_O emissions also in boreal peatlands of Northeast China (Cui et al., 2016).

Freshwater ecosystems have often been ignored in consideration of the landscape GHG fluxes. We used global warming potential (GWP; GWP_100_ = 265; Ciais et al., 2013) to estimate the effect of N_2_O evasion from Finnish lakes on the climate as CO_2_ equivalents. Our estimate (0.6 Gg N_2_O‐N/year; Table 4) represents 35% (the 25%–75% quartiles being 29%–41%) of the GWP of Finnish lake diffusive CH_4_ emissions (Juutinen et al., 2009). It is estimated that ebullition may even double the methane emissions (Bastviken, Cole, Pace, & Tranvik, 2004; Juutinen et al., 2009; Wik et al., 2014). When the uncertainty related to methane ebullition is taken into account (i.e., scenarios with and without ebullition included), our N_2_O estimate represents 15%–41% of the GWP of Finnish lake CH_4_ emissions. Assuming similar atmospheric impact for boreal lakes in general gives an emission estimate of 29 Gg N_2_O‐N/year.

Annual mean and median N_2_O flux estimates from our lakes including seasonal data were surprisingly close to each other (Table 5) while estimates based only on summer measurements underestimated annual N_2_O emissions (Table 4). Furthermore, the different annual N_2_O flux estimates resulting from the three approaches for gas transfer coefficients (*k* values) and their dependence on lake size (Heiskanen et al., 2014; Holgerson et al., 2017; Vachon & Prairie, 2013) demonstrate that there is uncertainty due to limited measured *k* values, especially for large lakes. Recently, Webb et al. (2019) showed widespread unexpected undersaturation (67%) of N_2_O in eutrophic farm water bodies in the United States. In our data, 29% of the samples were undersaturated, majority of these were sampled during summer.

Our data demonstrated large seasonal variation of nitrate and N_2_O in boreal lakes and the important role of winter in annual emission estimates, the neglect of which results in underestimation of annual N_2_O flux estimates. During recent mild winters, the ice cover period has been shorter and warm autumns have delayed the freezing day. Assuming 1 month shorter ice cover period (we extended autumn to 3 months, since especially autumn temperatures have been rising during recent years) resulted in 15% larger evasion estimates. Climate change scenarios predict increasing temperature and precipitation for northern Europe (Ciais et al., 2013) accompanied by increasing frequency of freeze–thaw events which have been shown to result in enhanced nitrate and N_2_O fluxes (Cui et al., 2016). Rising temperature has further been shown to result in earlier spring snow melt floods throughout northeastern Europe (Blöschl et al., 2017) which contributes to seasonal distribution of nitrate transport from land to lakes and further to the overall role of lakes as N_2_O sources in the boreal landscape.

## CONFLICT OF INTERESTS

The authors declare no competing financial interests.

## Data Availability

The data that support the findings of this study are available from the corresponding author upon reasonable request.
