# Supplementary material for: Lakes as nitrous oxide sources in the boreal landscape
Source: Glob Chang Biol. 2020 Jan 8;26(3):1432–45. doi: 10.1111/gcb.14928 (PMC7078959; doi:10.1111/gcb.14928)
Supplement: Supplementary file 1 [file GCB-26-1432-s001.pdf]

Supporting Information

Table S1. Estimates of annual N_2O flux (Gg N_2O-N y^{-1}) from Finnish and Boreal lakes. Using three approaches for estimating gas transfer velocity (Vachon & Prairie 2013, Heiskanen et al. 2014, Holgerson et al. 2017). Annual estimates were calculated from our flux data from the randomly selected lakes using different upscaling approaches. The estimates for each lake size class separately (by lake size class) and for all size classes combined (the mean, median and summer median of individual lakes) were multiplied by the area of the Finnish and boreal lakes. For the median flux, the 25% and 75% quartiles and for the mean flux 95% confidence intervals, respectively, are given in parentheses. Two small humic lakes with fluxes of 863 and 22 085 mg N_2O-N m^{-2} y^{-1} were excluded as outliers.

Holgerson et al. (2017)

Upscaling approach	Finnish Lakes	Boreal lakes
median flux by lake size class	0.6 (0.5-0.7)	29 (26-32)
median flux of individual lakes	0.3 (0.09-0.7)	12 (4-31)
summer median flux of individual lakes	0.07 (-0.2-0.3)	3 (-10-13)
mean flux of individual lakes	0.5 (0.3 -0.7)	23 (15-31)
Area (km^2)	32 663[1]	1 422 448[2]

Heiskanen et al. (2014)

Upscaling approach	Finnish Lakes	Boreal lakes
median flux by lake size class	0.6 (0.5-1.6)	28 (13-37)
median flux of individual lakes	0.4 (0.2-1)	19 (7-41)
summer median flux of individual lakes	0.1 (-0.3-0.4)	5 (-14-17)
mean flux of individual lakes	0.8 (0.5 -1)	33 (22-44)
Area (km^2)	32 663[1]	1 422 448[2]

Vachon and Prairie (2013)

Upscaling approach	Finnish Lakes	Boreal lakes
median flux by lake size class	0.8 (0.8-1.0)	40 (37-43)
median flux of individual lakes	0.4 (0.1-1)	19 (5-41)
summer median flux of individual lakes	0.1 (-0.3-0.4)	5 (-15-19)
mean flux of individual lakes	0.8 (0.5 -1)	33 (22-46)
Area (km^2)	32 663[1]	1 422 448[2]

[1]Lake size distribution[21]

[2]Lake size distribution estimated based on MODIS data, excluding lakes <0.1 km^2

Table S2. Annual N_2O flux estimates (mg N_2O-N m^{-2} y^{-1}) by lake type based on all those lakes that were sampled at four occasions (n=94; the water quality data were missing from two lakes and the lake type could not be assigned): Annual fluxes for the randomly selected lakes (n=71), the subset of Eutrophic lakes with the highest total P concentrations (n=23). The annual fluxes consist of fluxes at the thaw (0.5 months), in spring (1.5 months), in summer (3 months), and in autumn (2 months), total of 7 months of ice-free season. Two small humic lakes with fluxes of 863 and 22 085 mg N_2O-N m^{-2} y^{-1} were excluded as outliers.

Gas transfer velocity estimates from (Holgerson et al. 2017)

Lake type/group	Mean	Median	SD	CV%	N
Nutrient rich, calcareous	43	35	33.1	78	12
Clear water	9	11	11.7	126	14
Humic, large	60	41	44.2	74	7
Humic, small	18	8	29.7	164	59
All	23	11	32.2	142	94
Randomly selected	16	9	25.1	155	71
Eutrophic	43	35	42.6	99	23

Gas transfer velocity estimates from Heiskanen et al. (2014)

Lake type/group	Mean	Median	SD	CV%	N
Nutrient rich, calcareous	69	66	48.8	70	12
Clear water	11	13	11.6	110	14
Humic, large	56	38	42.2	76	8
Humic, small	25	12	38.9	156	59
All	31	16	41.3	135	94
Randomly selected	23	13	34.1	147	71
Eutrophic	54	38	53.9	101	23

Gas transfer velocity estimates from Vachon and Prairie (2013)

Lake type/group	Mean	Median	SD	CV%	N
Nutrient rich, calcareous	68	67	50.2	74	12
Clear water	13	13	15.2	120	14
Humic, large	83	54	58.1	70	7
Humic, small	26	12	41.1	161	59
All	33	16	45.6	138	94
Randomly selected	24	13	36.2	153	71
Eutrophic	61	49	59.0	96	23
